# Supplementary material for: Identifying Factors Predicting Kidney Graft Survival in Chile Using Elastic-Net-Regularized Cox’s Regression
Source: Medicina (Kaunas). 2022 Sep 26;58(10):1348. doi: 10.3390/medicina58101348 (PMC9608564; doi:10.3390/medicina58101348)
Supplement: Supplementary file 1 [file medicina-58-01348-s001.zip › medicina-1844559-supplementary.pdf]

Supplementary Material

Index:
1. Training algorithm
2. Missing value imputation
3. Exploratory data analysis
4. Kaplan-Meier survival curves
5. Descriptive statistics
6. Log-linearity analysis
7. Model Training
8. Assumption validation
9. Donor gender analysis

Supplementary Algorithms:
Algorithm S1: Training algorithm for variable selection and model fitting.

Supplementary Tables:
Table S1: Statistical comparison between imputed and preexisting values.
Table S2: Categories used for numeric variables in Kaplan-Meier analysis.
Table S3: Descriptive summary of recipient characteristics.
Table S4: Descriptive summary of donor and transplant characteristics.
Table S5: Transformations included as study variables.
Table S6: Proportionality assumption tests in each predictor and in the overall model.
Table S7. Comparisson of means and proportions between donor genders.

Supplementary Figures:
Figure S1: Exploratory data analysis.
Figure S2: Graphic representation of Spearman's correlation matrix.
Figure S3: Kaplan-Meier analysis in donor characteristics.
Figure S4: Kaplan-Meier analysis in recipient characteristics.
Figure S5: Preliminary log-linearity assumption analysis.
Figure S6: Log-linearity assumption analysis via penalized smoothing splines.
Figure S7: Proportionality assumption analysis via scaled Schoenfeld residuals.

1. Training Algorithm

Three conditions were considered when selecting the predictive model for transplant outcome. Firstly, the results must be comprehensible for the medical community in charge of discussing the organ allocation system in Chile. Secondly, it should objectively identify the predictors associated with transplant outcome, considering statistical evidence and estimate effects using available data. And thirdly, it must provide adequate flexibility to be applied in future studies as more national data is collected from transplant centers.

Taking these conditions into account, we designed a training algorithm based on a Cox Proportional Hazards regression regularized by the Elastic Net penalty. Two aspects of the methodology were considered for this decision. On one hand, the base form of the hazard function in Cox's model has been widely applied in literature and has been used by the Kidney Allocation System in USA (EPTS and KDPI scores), demonstrating to be a comprehensible metric for decision makers. On the other hand, the Elastic-Net regularization promote generalization of the mechanism and deliver an automated procedure to identify predictors related with transplant outcome from the variables at disposal.

For a robust training of the algorithm and considering our sample size (n=822) and the observed failures in groups of patients with relevant comorbidities, the Algorithm 1 was developed. This considers a penalization close to pure Lasso (with an alpha equal to 0.95) with the purpose of performing variable selection maintaining stability in presence of highly correlated variables. Nevertheless, to obtain a simplified model we consider the highest penalty with a C-Index that is no further than one standard deviation from the optimum as it is widely applied in literature. This procedure was repeated 1000 times with the purpose of reducing the randomness in group selection for cross validation and to facilitate robustness in the replication of results. Finally, the variables associated with nonzero coefficients in more than the half of the scenarios are defined as the identified predictors for graft failure.

Algorithm 1 Training algorithm for variable selection

Require: $N = 1000$, $k = 10$
  Initialize the counter for each variable in zero.
  for $i \in \{1, ..., N\}$ **do**
    Randomly divide data in k disjoint subsets.
    Be G the resulting set of k disjoint subsets.
    for $g \in G$ **do**
      Take g as testing data.
      Take $G/\{g\}$ as training data.
      Fit Cox Elastic Net model in training data for different values of λ.
      Compute C-Index for each value of λ in testing data and save.
      Save the C-Index obtained for each value of λ.
    end for
    Compute average C-Index for each value of λ.
    Be λ^* the value of λ related with the maximum average C-Index.
    Take the highest λ related to an average C-Index no further than 1 s.d. from λ^* (λ^{1se}).
    Increase counter for variables with non-zero coefficients in λ^{1se} by one.
  end for
  Label the variables with counter greater than $N/2$ as selected predictors.

The identified predictors were used as covariables in a multivariate Cox proportional hazard risk model to communicate confidence intervals for hazard ratios. Predictive accuracy was assessed using C-Index in the training sample as well as in an out of sample testing dataset, consisting of

76 kidney transplants omitted from the training because of inconsistent information in relevant variables assessed as potential predictors but not selected in the final model.

2. Missing Forest Imputation

Missing Forest algorithm was used to replace missing values with reasonable estimates in the following four variables: donor creatinine mg/dL (9.6%), recipient weight (6.8%), recipient years on dialysis (2.5%) and cold ischemia time (0.9%). The implementation was performed in Python (version 3.8.8) using the class *MissForest* from package *survival* with an arbitrary random state parameter equal to 4255. The remaining parameters were kept at default values and are detailed in the package reference manual. A statistical comparison between imputed values and preexisting ones is shown in the following Table S1. For missing values in donor history of diabetes (9.8%) the cases were revised, and the value was replaced by cero, i.e., the absence of the comorbidity.

Table S1: Statistical comparison between imputed and preexisting values.

Variable	Mean (SD)	Min	Max	Median (IQR)
Donor creatinine (mg/dL)				
Observed	0.87 (0.36)	0.10	3.19	0.80 (0.66-1.00)
Imputed	0.84 (0.17)	0.47	1.49	0.81 (0.74-0.88)
Recipient weight (kg)				
Observed	67.0 (12.7)	36.0	164.0	66.0 (58.0-75.0)
Imputed	66.5 (9.7)	44.5	84.0	68.9 (59.7-73.5)
Recipient years on dialysis				
Observed	4.9 (3.8)	0.0	25.2	4.0 (2.2-6.7)
Imputed	3.9 (2.1)	1.4	8.3	3.1 (2.4-5.1)
Cold ischemia time (hr)				
Observed	15.5 (9.5)	0.0	40.1	17.0 (10.9-23.0)
Imputed	18.9 (3.1)	15.1	23.5	18.3 (17.1-20.0)

3. Exploratory Data Analysis

Figure S1 shows three exploratory data subfigures. Subfigure (a) shows a visual description of the frequency of renal transplants by year and transplant center. It is observed that almost 91% of the data comes from three transplant centers. Subfigure (b) shows the density distribution of observed death-censored survival times. It can be observed that a relevant proportion of registered graft-failure times is observed in the short term; near 34% of total are registered during the first 6 months from the transplant. Finally, subfigure (c) shows the density distribution for censored follow-up times; being concentrated between the 4 and 10 years from transplant with a follow-up median time in 7.2 (IQR: 4.9–10.2) years from transplant.

On the other hand, Figure S2 shows a graphic representation for the Spearman's correlation coefficients between the variables in study. The size of each circle represents the absolute value of the effect (as well as the intensity of the color) and the color indicates the sign of the correlation (blue represents a positive correlation and red negative). Finally, we assessed multicollinearity among the model features computing the Variance Inflation Factor for each variable, all of which yield values below 1.5, suggesting this is not an issue in the analysis.

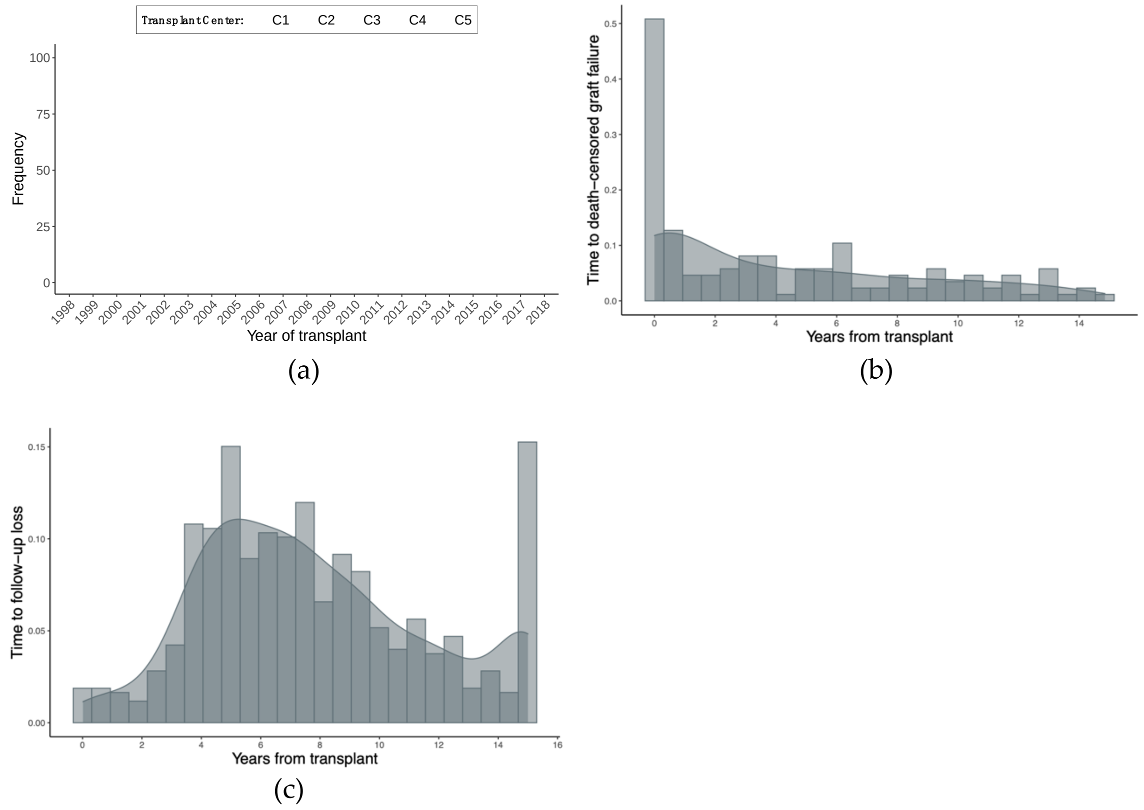

Figure S1. Exploratory data analysis showing: (a) Number of transplants in study per year and transplant center; (b) density distribution of observed death-censored graft failure times; and (c) density distribution of censored follow-up loss times.

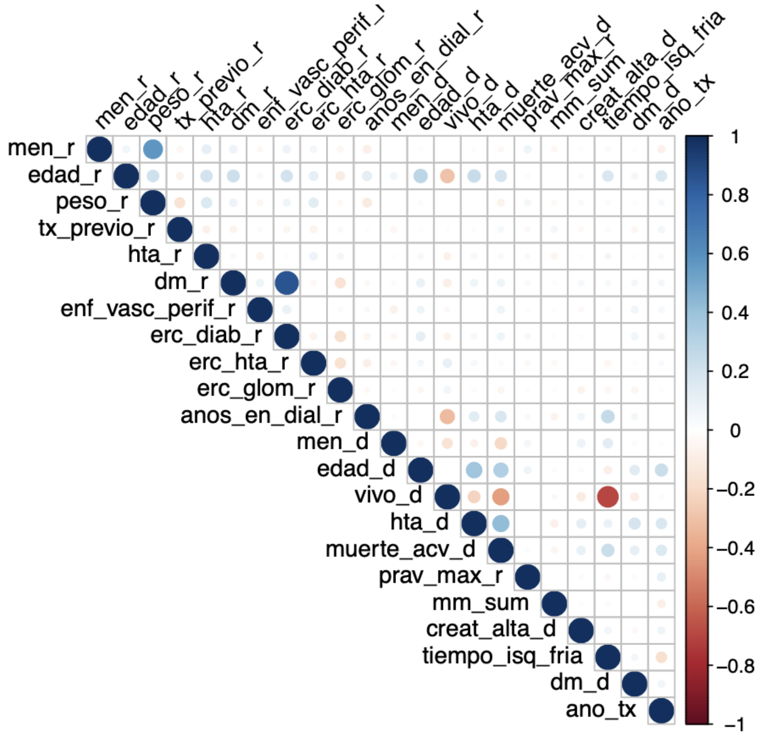

Figure S2. Graphic representation of Spearman's correlations coefficients between transplants characteristics in study.

Calculated Variance Inflation Factor for the selected variables:

Variable	VIF
Donor Male Sex	1.02
Recipient Previous Tx	1.01
Donor Age	1.22
Recipient ln(Years on Dialysis + 1)	1.03
Donor Diabetes	1.07
Donor Hypertension	1.25
Mismatch HLA	1.03

4. Kaplan-Meier Survival Curves

The Kaplan-Meier estimator is used to estimate the survival function in different groups of patients for each study variable. This is done with the purpose of collecting preliminary non-parametric evidence to be compared with the algorithm results. Each Kaplan-Meier plot shows the estimated evolution of the graft survival probability in time. Furthermore, the *logrank* test is used to check whether there exists significant difference between the survival curves. The resulting p-value is presented in the left-bottom quadrant in each plot. Finally, for numeric variables, the categories detailed in Table S2 were considered based on statistical and medical criteria.

Table S2. Categories used for numeric variables in Kaplan-Meier analysis.

Variable	N° Groups	Categories
Recipient Age	3	18-38; 39-50; 51-75.
Donor Age	3	18-38; 39-50; 51-79.
Recipient Years on Dialysis	3	0-2 years; 2-4 years; >4 years.
Recipient Max PRA	3	0-10 %; 11-50 %; 51-100 %.
Mismatch HLA	3	0-1 MM; 2-3 MM; 4-6 MM.
Year of Transplant	3	1998-2005; 2006-2010; 2011-2018.

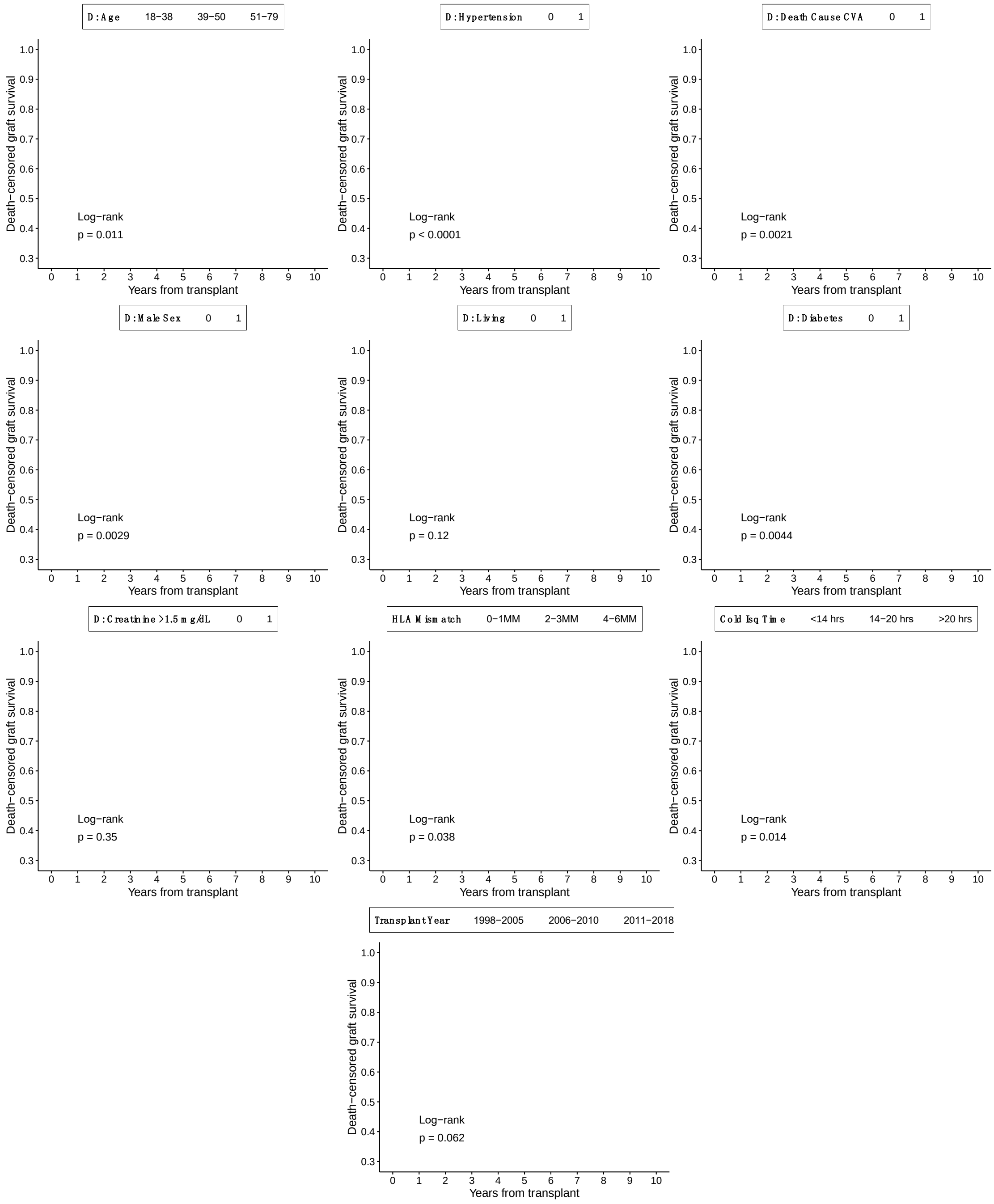

Figure S3. Kaplan-Meier analysis in donor characteristics. The vertical axis represents the estimated death-censored survival probability and the horizontal axis the time from the transplant in years.

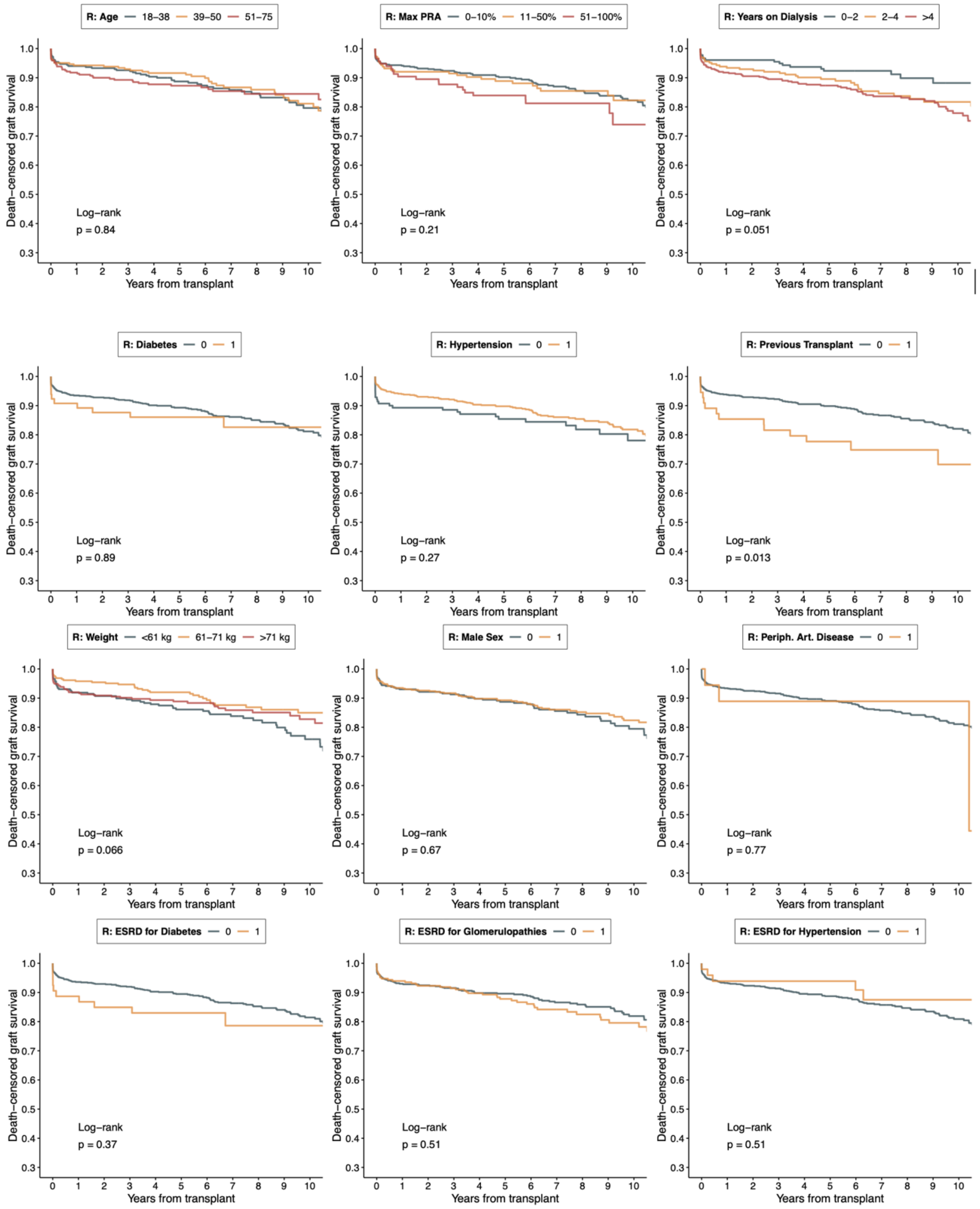

Figure S4. Kaplan-Meier analysis in recipient characteristics. The vertical axis represents the estimated death-censored survival probability and the horizontal axis the time from the transplant in years.

5. Descriptive statistics

Table S3 shows a statistical description for recipient's characteristics. Most renal transplants were performed to recipients with history of Hypertension (83%), and with a maximum PRA not greater than 10% (64% of patients). Additionally, the average recipient presents an age near 45 years old, a weight of 67 kilograms, and has spent 3 years on dialysis approximately. Also, there exist minor groups of patients with medical history of relevant comorbidities: 65 (8%) patients with Diabetes Mellitus, 18 (2%) with peripheral arterial disease, and others.

Table S3. Descriptive summary of recipient characteristics. In bold are shown the p-values significant at a 25% significance level in Cox univariate regression.

			**Univariate Cox Regression**	
Variable	Mean (SD) / n(%)	Events	Hazard Ratio (95% CI)	p-value
Recipient: Age	44.62 (12.91)		1.00 (0.99 - 1.01)*	> 0.9
Recipient: Weight	66.78 (12.62)		0.99 (0.98 - 1.01)*	**0.2**
Recipient: Years on Dialysis	3.16 (2.78)		1.05 (0.99 - 1.11)*	**0.10**
Recipient: Male sex				
Yes	463 (56%)	81	0.94 (0.68 - 1.30)	0.7
No	359 (44%)	64	1.00	
Recipient: Previous Transplant				
Yes	55 (7%)	15	1.87 (1.09 - 3.19)	**0.035**
No	767 (93%)	130	1.00	
Recipient: Diabetes Mellitus				
Yes	65 (8%)	11	1.14 (0.61 - 2.10)	0.7
No	757 (92%)	134	1.00	
Recipient: Hypertension				
Yes	682 (83%)	113	0.74 (0.50 - 1.10)	**0.2**
No	140 (17%)	32	1.00	
Recipient: Periph. art. disease				
Yes	18 (2%)	3	1.19 (0.38 - 3.73)	0.8
No	804 (98%)	142	1.00	
Recipient: ESRD cause DM2				
Yes	53 (6%)	11	1.44 (0.78 - 2.67)	0.3
No	769 (94%)	134	1.00	
Recipient: ESRD cause HTA				
Yes	49 (6%)	6	0.74 (0.33 - 1.69)	0.5
No	773 (94%)	139	1.00	
Recipient: ESRD cause Glom.				
Yes	248 (30%)	49	1.17 (0.83 - 1.65)	0.4
No	574 (70%)	96	1.00	
Recipient: Maximum PRA				
0-10%	529 (64%)	96	1.00	
11-50%	177 (22%)	27	0.84 (0.55 - 1.29)	0.4
51-100%	116 (14%)	22	1.35 (0.85 - 2.12)	**0.2**

Table S4 shows a statistical description for the transplant and donor characteristics in study. Most of kidneys come from cadaveric donors (80%), male sex (58%), and without history of Hypertension (81%). The 88% of the transplants present at least 2 HLA Mismatch donor-recipient and the average donor is approximately 44 years old (IQR: 34–55).

Table S4. Descriptive summary of donor and transplant characteristics. In bold are shown the p-values significant at a 25% significance level in Cox univariate regression.

			Univariate Cox Regression	
Variable	Mean (SD) / n(%)	Events	Hazard Ratio (95 % CI)	p-value
Donor: Age	43.88 (13.03)		1.03 (1.01 - 1.04)*	**<0.001**
Transplant: Cold ischemia time	15.62 (9.52)		1.01 (1.00 - 1.03)*	**0.12**
Donor: Male sex				
Yes	475 (58 %)	71	0.65 (0.47 - 0.89)	**0.009**
No	347 (42 %)	74	1.00	
Donor: Living				
Yes	164 (20 %)	22	0.71 (0.45 - 1.12)	**0.12**
No	658 (80 %)	123	1.00	
Donor: Hypertension				
Yes	158 (19 %)	39	2.24 (1.54 - 3.25)	**<0.001**
No	664 (81 %)	106	1.00	
Donor: Diabetes				
Yes	26 (3 %)	8	2.72 (1.33 - 5.57)	**<0.017**
No	796 (97 %)	132	1.00	
Donor: Creatinine > 1.5 mg/dL				
Yes	36 (4 %)	7	1.44 (0.67 - 3.08)	0.40
No	784 (96 %)	133	1.00	
Donor: Cause of death CVA				
Yes	346 (42 %)	69	1.67 (1.20 - 2.33)	**0.002**
No	476 (58 %)	76	1.00	
Transplant: HLA Mismatch				
0-1MM	95 (12 %)	10	1.00	0.3
2-3MM	445 (54 %)	81	1.71 (0.89 - 3.31)	**0.10**
4-6MM	282 (34 %)	54	2.12 (1.08 - 4.18)	**0.028**

Univariate Cox regressions were adjusted in each variable to investigate associations with death-censored graft failure under the model assumptions independently. The results are shown in Tables S4 and S5, where p-values are shown in bold as a 25% significance level was considered for detecting potential associations as Bursac et al. (2008) suggest.

6. Log-linearity analysis

The log-linearity assumption is preliminarily studied to establish functional transformations for a better fit in the Cox's hazard function modelling when evidence suggested. For this purpose, penalized smoothing splines with fives degrees freedom were fitted in univariate Cox regressions as it's suggested by Amini (2015). The implementation was performed in R (version 4.1.2) using the function *pspline* from the package *survival*.

Figure S5 shows the fitted penalized smoothing splines for each of the six non-binary study variables. Each plot shows the smoothed effect for the relation between the variable and the log-hazard, being a straight line the perfect adjustment for the log-linearity assumption. The significance of non-linear association was studied analyzing graphical evidence as well as testing the significance of the non-linear component of the spline to explain the hazard function. The test p-value is shown in the top-left corner of each plot.

Results indicate a good fit of the log-linearity assumption except for recipient age, where a significant non-linear association is observed. The graphical adjustment indicates an

increment in log-hazard for each year beyond 50 years old approximately (see Figure S5a). Therefore, it is determined to include a transformation to contemplate an effect for every year in the recipient beyond 50 years old. In addition, from analyzing the graphical adjustment three more transformations are proposed and included as study variables. The four transformations incorporated are shown in the following Table S5.

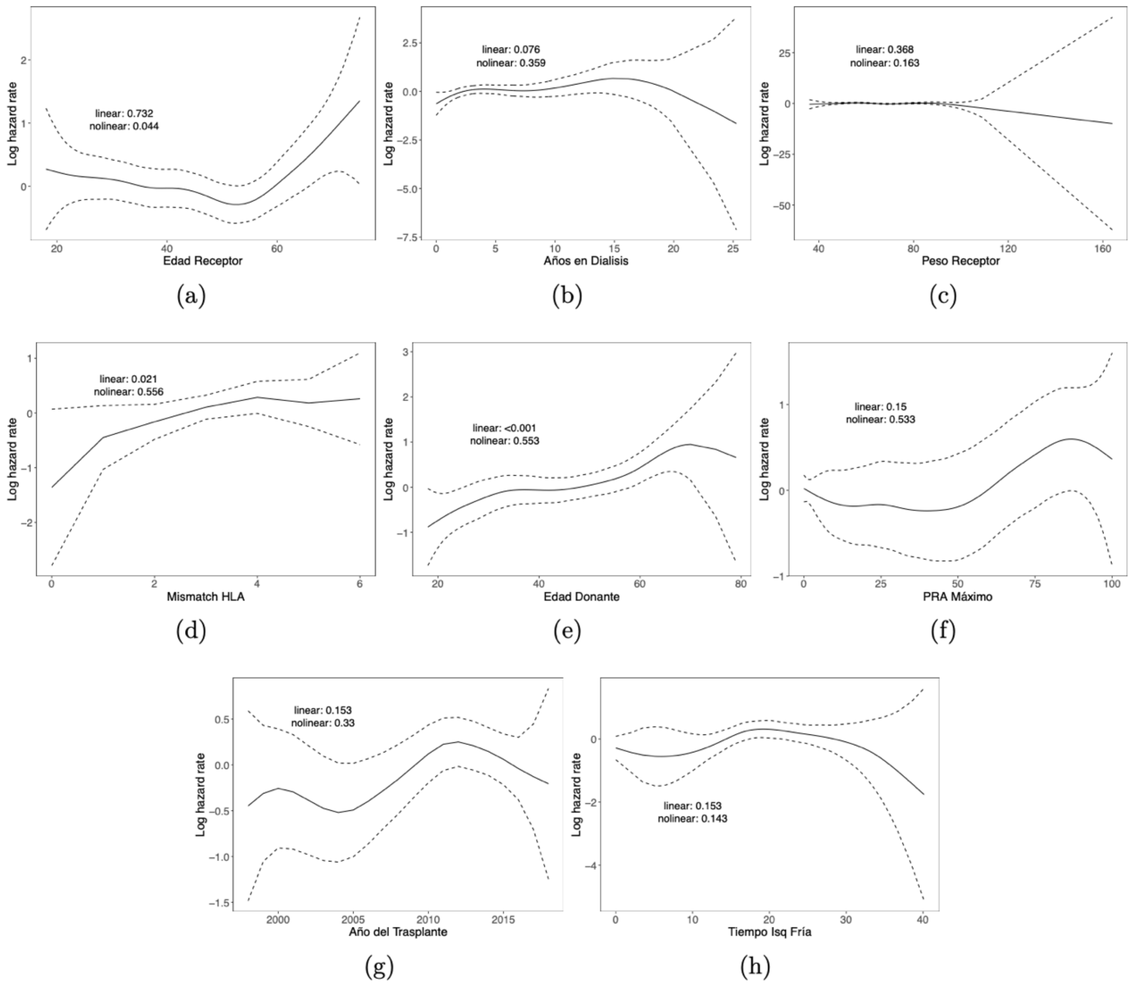

Figure S5. Preliminary log-linearity assumption analysis. Penalized splines with 5-degrees freedom were fitted in Cox univariate regressions for eight non-binary variables: (a) Recipient age, (b) Recipient years on dialysis, (c) Recipient weight, (d) HLA Mismatch, (e) Donor age, (f) Recipient maximum PRA, (g) Year of transplant, and (h) Cold ischemia time. In each plot the p-values for testing associations with hazard rate are presented.

Table S5. Transformations included as study variables.

New variable	Transformation	Description
$edad\_r\_desde\_50$	$max\{edad\_r - 50, 0\}$	Recipient years old beyond 50.
$pra\_max\_r\_gre\_50$	$I(pra\_max\_r > 50)$	Maximum PRA grater than 50 %.
$log\_anos\_dial\_r$	$ln(anos\_dial\_r + 1)$	Log transformation years on dialysis.
log_peso_r	$ln(peso\_r + 1)$	Log transformation recipient weight.

7. Model Training

The training procedure was implemented in R (version 4.1.2) using the function *cv.glmnet* from the package *glmnet* (see package documentation) with parameters *alpha* equal to 0.95, *nfolds* equal 10 and *type-measure* referencing C-Index. This uses the algorithm proposed by Simon et al. (2011) which fits via cyclical coordinate descent and employs warm starts to find a solution along the regularization path efficiently. Also, before the execution, an arbitrary selected random seed equal to 1234 was set for reproduction of the results.

8. Assumption validation

In the first place, to evaluate log-linearity assumption, penalized smoothing splines with 5-degrees freedom were adjusted independently in every non-binary predictor to evaluate functional relations with the log-hazard. The significancy for the relation between the non-linear component and the risk of graft failure was evaluated at a 5% significance level and complemented with graphical evidence.

The implementation was performed in R (version 4.1.2) using the function *pspline* from the package *survival*. Figure S7 show the adjustments and, in the top left quadrant, the p-values for the test over the non-linear component. Results indicate a good adjustment of the assumption. The non-linear components are not significant with p-values of 0.584 for donor age; 0.422 for HLA Mismatch; and 0.284 for recipient years on dialysis log-transformation.

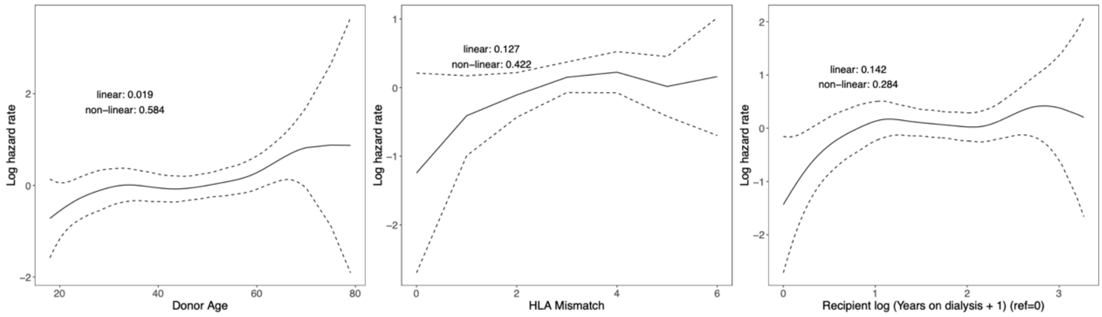

(a) (b). (c)

Figure S6. Log-linearity assumption analysis via 5-degrees freedom penalized smoothing splines adjusted independently for the three non-binary variables in the model: (a) donor age, (b) HLA mismatch and (c) years on dialysis log-transformation.

In the second place, for evaluating the proportionality assumption, the scaled Schoenfeld residuals were computed and analyzed. Specifically, for every predictor we obtained a smoothed graph that estimates the evolution of the coefficient in time by smoothing the sum between the scaled residuals and the estimated coefficient in the Cox model. This corresponds to $\hat{\beta}(t) = \widehat{V_k^{-1}r_k} + \hat{\beta}$ for each event time t_k, where $\widehat{V_k^{-1}r_k}$ represents the scaled Schoenfeld residual for the variable in analysis in the event time t_k. The null hypothesis that the proportionality assumption is met was tested applying the statistic χ^2 distributed proposed by Grambsch and Therneau (1994) and was evaluated at a 5% significance level.

The implementation was coded in R (version 4.1.2) using the function *cox.zph* from the package *survival.* Table S6 shows the resulting p-value for the proportionality test in each predictor as well as the general goodness of fit test of the assumption. The results show that there is not statistically significant evidence to reject the null hypothesis in the overall model (p-value equal to 0.4) as well as in every predictor (the smallest p-value equals to 0.093 for HLA mismatch and it's complemented with graphical evidence). Figure S7 show the smoothed estimation for the evolution of the coefficient in time, and looks approximately like a random walk near zero, which indicates that the assumption is reasonable.

Table S6. Proportionality assumption test in each predictor and overall goodness of fit test for the model using the statistic detail by Grambsch and Therneau (1994).

Variable	Chisq	df	p-value
Donor Hypertension (ref=No)	1.415	1	0.234
Donor Age (ref=40)	0.254	1	0.613
Previous transplant (ref=No)	0.456	1	0.499
Donor Male sex (ref=Female)	1.151	1	0.283
R: ln(Years on dyalisis + 1) (ref=0)	0.731	1	0.392
HLA Mismatch (ref=0MM)	2.824	1	0.093
Donor Diabetes (ref=No)	0.135	1	0.713
Global	7.282	7	0.400

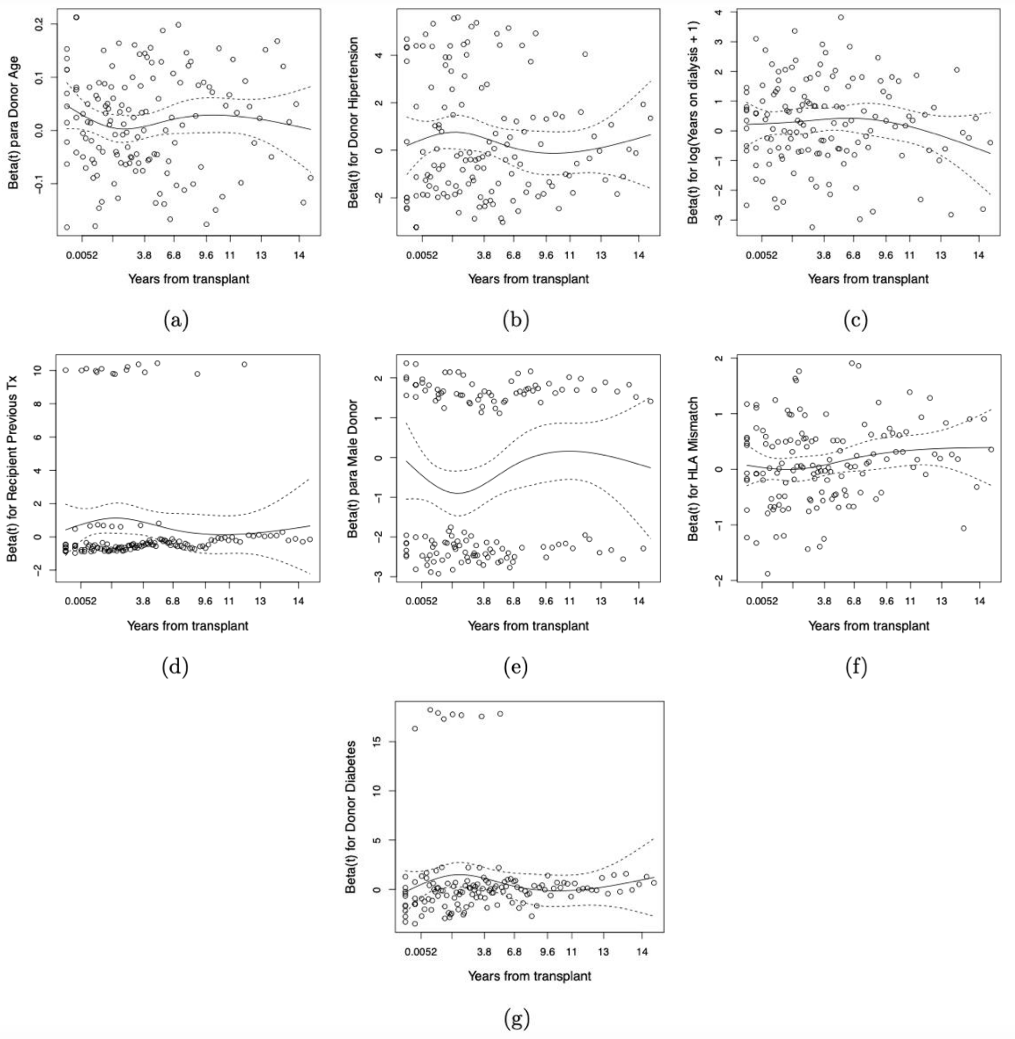

Figure S7. Graphical proportionality assumption analysis via the smoothed distribution of scaled Schoenfeld residuals over event times.

9. Donor gender analysis

Table S7. Comparisson of means and proportions between donor gender.

Variable	Donor Gender		p-value[1]
	**Female (N=347)**	**Male (N=475)**	
**Donor Weight (kg)**			<0.001
Mean (SD)	66.34 (10.76)	77.53 (9.90)	
Median (IQR)	65.00 (60.00-70.75)	79.00 (70.00-85.00)	
Range	42.00, 103.00	48.00, 110.00	
Unknown	241	308	
**Donor Height (cm)**			<0.001
Mean (SD)	159.11 (6.86)	171.15 (6.34)	
Median (IQR)	160.00 (155.00-164.75)	170.00 (168.50-175.00)	
Range	144.00, 183.00	145.00, 190.00	
Unknown	241	309	
**Donor BMI (kg/m^2)**			0.110
Mean (SD)	25.82 (4.05)	26.07 (3.02)	
Median (IQR)	25.00 (23.00-27.00)	26.00 (24.00-28.00)	
Range	18.00, 39.00	17.00, 39.00	
Unknown	242	309	
**Donor Death by CVA**			<0.001
Yes	159/347 (46%)	317/475 (67%)	
No	188/347 (54%)	158/475 (33%)	

[1] Wilcoxon rank sum test; Pearson's Chi-squared test.
